# A coiled‐coil domain triggers oligomerization of MmpL10, the mycobacterial transporter of trehalose polyphleate precursor

**DOI:** 10.1002/1873-3468.70085

**Published:** 2025-06-04

**Authors:** Julie Couston, Jérôme Feuillard, Aurélie Ancelin, Joséphine Lai‐Kee‐Him, Konstantin Brodolin, Christian Chalut, Pontus Gourdon, Mickaël Blaise

**Affiliations:** ^1^ Institut de Recherche en Infectiologie de Montpellier (IRIM) Montpellier University, CNRS France; ^2^ Centre de Biologie Structurale (CBS) Montpellier University, CNRS, INSERM France; ^3^ INSERM, Institut de Recherche en Infectiologie Montpellier France; ^4^ Institut de Pharmacologie et de Biologie Structurale (IPBS) Université de Toulouse, CNRS, Université Toulouse III – Paul Sabatier (UT3) France; ^5^ Department of Experimental Medical Science Lund University Sweden; ^6^ Department of Biomedical Sciences Copenhagen University Denmark

**Keywords:** coiled‐coil, MmpL, mycobacteria, *Mycobacterium smegmatis*, RND transporter, TPP

## Abstract

The mycobacterial outer membrane is composed of unusual lipids and glycolipids. Some of these lipids are exported to the cell envelope by resistance‐nodulation‐division (RND) transporters called mycobacterial membrane protein large (MmpL). While the oligomeric state of most RND transporters is well established, MmpL assembly remains unclear. Here, we investigated MmpL10, the trehalose polyphleate transporter. Biochemical data suggest that MmpL10 forms a homotrimer and that its oligomerization is driven by a coiled‐coil domain. Structural modeling and electron microscopy data reveal the presence of a tubular extension that spans the mycobacterial cell wall and reaches the mycomembrane. As most MmpL proteins possess this extension, oligomerization may be a common feature of this family of transporters, possibly involved in the transport of the MmpL cargo.

## Graphical abstract

Most MmpLs transport lipids and glycolipids of the complex mycomembrane of mycobacteria. This study shows that MmpL10 from *Mycobacterium smegmatis*, the transporter of the trehalose polyphosphate precursor, harbors a coiled‐coil‐like extension. Biochemical and electron microscopy studies demonstrate that the coiled‐coil enables MmpL10 to trimerize. The extension potentially forms a channel that transports glycolipids through the thick cell wall of mycobacteria. The figure was generated with chimerax and inkscape.

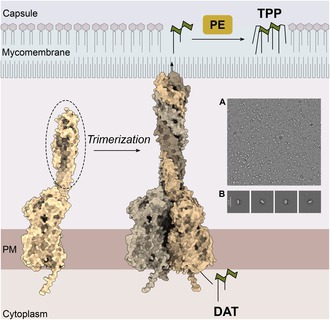

## Abbreviations

**CC**, coiled‐coil

**EM**, electron microscopy

**LMNG**, lauryl maltose neopentyl glycol

**MmpL**, mycobacterial membrane protein large

**Msm**, *Mycobacterium smegmatis*

**MW**, molecular weight

**SEC**, size‐exclusion chromatography

**TPP**, trehalose polyphleate

Mycobacteria possess an atypical cell envelope made of a plasma membrane, a peptidoglycan layer, an arabinogalactan layer, an outer membrane, called mycomembrane, composed of layers of highly complex lipids and glycolipids, and a capsule. Some of these lipids and glycolipids have up to 90 carbon atoms in their aliphatic chains that are end products of complex biosynthetic pathways. Many of the enzymes involved in the synthesis of these complexed compounds have already been discovered and are well characterized, and represent attractive targets for the development of new drugs to treat mycobacterial infections. Outer membrane lipids and glycolipids, or their precursors, are synthesized in the cytoplasm and must be flipped over the plasma membrane and then transported through the cell wall to reach their final destination. To date, little is known about the mechanisms and molecular machineries involved in the export and assembly of outer membrane lipids in mycobacteria.

Previous studies have shown that a subclass of resistance‐nodulation‐division (RND) transporters, called mycobacterial membrane protein large (MmpL), contributes to the transport of mycomembrane lipids and other molecules, such as siderophores in mycobacteria. Depending on the mycobacterial species, up to 31 genes encoding MmpLs can be found [1]. MmpL3, the only MmpL essential for mycobacterial viability, transports trehalose monomycolate (TMM) [2] and is considered a very promising target to combat mycobacterial infections, including Tuberculosis. The MmpL3 crystal structure [3, 4] revealed the presence of 12 transmembrane helices divided into two transmembrane domains, each harboring a periplasmic domain, like most other members of the RND family. MmpL3 flips TMM over the plasma membrane before the cargo is transported across the cell wall by a yet‐to‐discover transport system. Biochemical investigations [5, 6, 7] have shown that MmpL3 flipping activity is energized, as for the other members of the RND family, by the proton motive force. MmpL3 X‐ray and cryo‐EM structures have shown that the protein can exist as a monomer [3, 4, 8, 9, 10], which is also the case for MmpL5 and MmpL4 from *Mycobacterium smegmatis* (*Msm*), both involved in the efflux of siderophores [11, 12]. However, the oligomeric status of MmpLs remains a controversial issue, as other studies revealed that MmpL3 exists both as a monomer and as a homodimer in solution [13]. In addition, biochemical and structural studies have shown that CmpL1, the corynebacterial orthologue of MmpL3, is a homotrimer [14], whereas single‐molecule and high‐resolution microscopy approaches have demonstrated that MmpL5 from *Mycobacterium tuberculosis* (*Mtb*) can exist *in vivo* as a homotrimer and that this oligomerization is enhanced by its binding partner MmpS5 [15]. More recently, structural and biochemical studies of MmpL4 identified potential oligomeric forms of the protein, but the stoichiometry of the oligomeric form could not be determined [16]. Indeed, several key defining members of the RND superfamily, including the multidrug exporter AcrB, operate as homotrimers [17].

MmpL10, albeit less studied, has gained recent interest as it transports the precursor of trehalose polyphleate (TPP) in numerous nontuberculous mycobacteria, including *Msm* and *Mycobacterium abscessus* (*Mab*). TPP are complex cell surface‐exposed glycolipids sharing related structures and biosynthetic pathways with diacyl/polyacyl‐trehaloses and sulfolipids from *Mtb*. TPP has recently been shown to be an essential component for the successful infection of *Mab* by some mycobacteriophages [18, 19]. Interestingly, TPP seems to play a role in the virulence of *Mab*, as a *Mab mmpL10* mutant is moderately impaired in its virulence in a zebrafish embryo model of infection [20].

The TPP biosynthesis pathway [21, 22] is initiated in the cytoplasm with the formation of a 2,3‐diacyltrehalose intermediate (hereafter referred to as DAT) bearing a C14–C19 fatty acyl group and a phleic acyl group. Following the export of DAT to the cell envelope, a transacylase, called PE, catalyzes the transesterification of phleic acids between DAT to generate TPP. Deletion of *mmpL10* in both *Msm* and *Mab* completely abolishes TPP production and results in the accumulation of DAT in the mutant strains, consistent with the role of this transporter in DAT translocation across the plasma membrane [21, 22]. How DAT is exported to its final destination to be the substrate of PE remains to be clarified, as no MmpL10‐protein partners have been identified.

In this context, we initiated a biochemical study to better understand how MmpL10 transports the TPP precursor. We report the recombinant expression and purification of MmpL10 (MSMEG_0410) from *Msm* (MmpL10_Msm_). By combining biochemical, structural modeling, and electron microscopy approaches, we demonstrated that MmpL10 forms oligomers in solution and that this oligomerization is dependent on a coiled‐coil extension of the periplasmic domain of MmpL10. Finally, we provide evidence that such an organization can be shared by most MmpLs.

## Materials and methods

### Cloning

The MmpL10‐mEGFP‐FL_Msm_ nucleotide sequence was synthesized (GenScript Biotech BV, Leiden, Netherlands) with codons optimized for *E. coli* expression. The construct comprised the whole *MSMEG_0410* sequence fused to one of mEGFP inserted into the pET‐15b backbone to give pET15::MmpL10‐mEGFP‐FL_Msm_. A *Strep*‐tag II motif was further added. The MmpL10‐mEGFP‐∆CC_Msm_ construct was obtained by site‐directed mutagenesis using the Q5 Site‐Directed Mutagenesis Kit (New England BioLabs, Evry, France) with the following primers: Fw 5′‐AACATCCCGCCGGAAGTGTTCGGTCTGGAAGACTTTAAGAAAG‐3′ and Rv 5′‐ACGGAATTCCGGCGGCACCTCACCCAGCGGACGGGTAATGCCGCT‐3′.

The expression plasmid for expressing the coiled‐coil domain alone (CC_Msm_) was obtained by polymerase chain reaction (PCR) from the full‐length MmpL10 plasmid, using the following primers: Fw 5′‐GGTACCGAGAACCTGTACTTCCAGGGTCGTGCGACCTTTCAAGCGGGTATTGTTGGCGACCG‐3′ and Rv 5′‐GGCTGAATTCCTCGAGTTACGGGTCCGCCGCGTTGTTACGCATGGTCAGCAGG‐5′. The PCR product was then cloned into the linearized Champion pET‐SUMO vector (Invitrogen) following the manufacturer's instructions. Finally, the MmpL10‐FL_Msm_ and MmpL10‐∆CC_Msm_ constructs were obtained by performing Q5 Site‐Directed Mutagenesis using the above‐described MmpL10‐mEGFP‐FL_Msm_ and MmpL10‐mEGFP‐∆CC_Msm_ and the following primers: Fw 5′‐ GGTAGCGGCAGCGGTAGCGGCGGTCATCACCACCATCACCATC‐3′ and Rv 5′‐ACCCTGGAAGTACAGGTTTTCGGTCACTTCCGCCGGCGCACGGCTG‐3′.

### Sample preparation optimization

Three different conditions for recombinant MmpL10‐mEGFP‐FL_Msm_ expression in *E. coli* C43 ∆*acrB* were tested in 100 mL Luria‐Bertani (LB) cultures supplemented with 200 μg·mL^−1^ ampicillin (Amp; Sigma‐Aldrich, Saint‐Quentin Fallavier, France) and 1 mm isopropyl‐ß‐d‐thiogalactopyranoside (IPTG; NeoBiotech, Nanterre, France): 3 h or overnight at 37 °C, and overnight at 18 °C after 30 min of cold shock on ice. Bacteria were collected, lysed by sonication, centrifuged, and proteins present in the supernatant were charged on a 10% SDS/PAGE gel to compare the expression and solubility levels of MmpL10‐mEGFP‐FL_Msm_ in the crude extract (CE) and the supernatant (SN); the fluorescence was measured with a Li‐Cor scanner using odyssey software and quantified with imagej.

The detergent solubility assay was performed with the following detergents: *n*‐dodecyl‐β‐d‐maltoside (DDM), *n*‐decyl‐beta‐maltoside (DM), lauryl maltose neopentyl glycol (LMNG), lauryldimethylamine‐N‐oxide (LDAO), n‐octyl‐β‐d‐glucoside (OG), octaethylene glycol monododecyl ether (C12E8), and Triton‐X100 (TX‐100); as well as sodium dodecyl sulfate (SDS) as a positive control. All the detergents were tested at 1% (w/v) except for C12E8, which was at a final concentration of 0.5% (w/v). Membranes were isolated by ultracentrifugation for 2 h 30 min at 185 000 ***g*** and 4 °C (Optima XE ultracentrifuge; Beckman Coulter, Villepinte, France) in a Ti 50.2 rotor, and resuspended by Dounce homogenization. The solution was then split and incubated with each detergent for 2 h at 4 °C, enabling the extraction of the proteins from the membranes. Each solution was further ultracentrifuged again for 1 h 30 min in the same parameters. The solubilized proteins collected in the supernatant were visualized and quantified according to the fluorescence levels of each detergent condition before and after ultracentrifugation. Measurement of fluorescence was performed with a Li‐Cor scanner using odyssey software and quantified with imagej. The efficiency of protein solubilization for each detergent was obtained by calculating the protein ratio in solution before and after ultracentrifugation.

### MmpL10‐mEGFP‐FL_Msm_ expression and purification

The pET15::MmpL10‐mEGFP‐FL_Msm_ was transformed into electrocompetent *E. coli* C43 ∆*acrB*. One clone was selected to start a 1.2 L preculture in LB culture supplemented with Amp (200 μg·mL^−1^). This preculture was used to inoculate a 36 L LB culture divided into 12 flasks. Bacteria were grown until OD_600_ reached 0.7–0.8, and protein expression was induced by adding 1 mm IPTG after a 30 min cold shock in an ice bath, and the culture was grown overnight at 18 °C. Bacteria were pelleted by a 15 min centrifugation at 8.983 ***g*** and 4 °C in a JLA‐8.100 rotor (Beckman Coulter), resuspended in 360 mL of lysis buffer (50 mm Tris‐HCl pH 8, 150 mm NaCl, 1 mm benzamidine, 10% glycerol, 0.5 mm β‐mercaptoethanol), and stored at −20 °C. For the purification, the solution of resuspended bacteria was slowly thawed in water and supplemented with a tablet of EDTA‐free Protease Inhibitor Cocktail (Sigma‐Aldrich, Saint‐Quentin Fallavier, France) and then sonicated four times during 1 min 30 (2 s pulse +2 s pause) at 40% intensity (Digital Sonifier; Branson Ultrasons SAS, Rungis, France). Lysed bacteria were centrifuged for 45 min at 27 216 ***g*** and 4 °C in a JA‐20 rotor (Beckman Coulter). The supernatant was collected and ultracentrifuged 2 h 30 min at 185 000 ***g*** and 4 °C (Optima XE ultracentrifuge; Beckman Coulter) in a Ti 50.2 rotor, and membranes were kept on ice overnight. The following day, the membranes were resuspended by Dounce homogenization in lysis buffer and the solution was diluted to 200 mL. Proteins were extracted and solubilized in 1% (w/v) LMNG for 2 h under stirring at 4 °C, and the solution was ultracentrifuged a second time in the same conditions for 1 h 30 min. The supernatant was incubated for 2 h with 4 mL *Strep*‐Tactin™ Superflow™ High‐Capacity Resin (IBA Lifesciences GmbH, Göttingen, Germany) previously equilibrated with lysis buffer, and the mix was then charged twice into two gravity columns. Beads were washed with 10 column volumes (CV) of lysis buffer supplemented with LMNG at 3× the critical micelle concentration (CMC, 0.003%), followed by a second wash of 25 CV of Tris‐HCl pH 8, 1 M NaCl, 10% glycerol, 0.5 mm β‐mercaptoethanol, 3× CMC LMNG. Beads were then incubated for 1 h with three CV of elution buffer [50 mm Tris‐HCl pH 8, 200 mm NaCl, 10% glycerol, 0.5 mm β‐mercaptoethanol, 3× CMC LMNG, and 2.5 mm d‐Desthiobiotin (Sigma‐Aldrich)]. The column was open and one last CV of elution buffer was added, and the solution was kept overnight at 4 °C. The day after, the solution was concentrated in a 100 kDa cutoff Centricon (Sartorius SAS, Palaiseau, France) until the final volume reached 1 mL. The solution was then charged on a size‐exclusion chromatography (SEC) on a Superose 6 Increase 10/300 GL column (Cytiva, Europe GMBH, Saint‐Germain en Laye, France) plugged on an ÄKTA go™ system (Cytiva). The protein was eluted at a flow rate of 0.35 mL·min^−1^ in 50 mm Tris‐HCl pH 8, 150 mm NaCl, and 3× CMC LMNG, with a fractionation volume of 250 μL in a 96‐deep well plate. Fifty microliters of each fraction were transferred to a new 96‐well plate, and fluorescence was measured by Tecan Infinite 200Pro apparatus using the Tecan i‐control v.2.0.10.0 software, measuring bottom fluorescence at excitation and emission wavelengths of, respectively, 485 and 535 nm.

This same protocol was used for the expression and purification of MmpL10‐mEGFP‐FL_Msm_ in LDAO and DDM detergents and at the same CMC as for LMNG. The MmpL10‐mEGFP‐∆CC_Msm_ was also purified similarly with the only difference that it was expressed overnight at 37°C and further eluted on a Superose 6 Increase 10/300 GL column or on a Superdex 200 Increase 10/300 GL columns (Cytiva). Similarly, MmpL10‐FL_Msm_ and MmpL10‐∆CC_Msm_ were also purified using this same protocol, and the presence of the protein was confirmed by western blot using an anti‐*Strep*‐tagII primary antibody and revealed with an anti‐mouse horseradish peroxidase secondary antibody (Sigma‐Aldrich).

### Size‐exclusion chromatography calibration curves

The apparent molecular weight (MW) of purified proteins was calculated after calibration of the Superose 6 Increase 10/300 GL column (Cytiva), injecting 1 mL of the following standard proteins (all from Sigma‐Aldrich) previously diluted in 1× phosphate‐buffered saline: thyroglobulin from bovine thyroid (670 kDa), β‐Amylase from sweet potato (200 kDa), bovine serum albumin (BSA, 66 kDa), and carbonic anhydrase from bovine erythrocytes (29 kDa). Successive runs were performed at a flow rate of 0.35 mL·min^−1^ in 50 mm Tris‐HCl pH 8, 150 mm NaCl, and proteins were eluted at 13.83, 16.80, 17.65, and 19.33 mL, respectively. The calibration of the Superdex 200 Increase 10/300 GL column (Cytiva) was performed similarly by using the following standard proteins: apoferritin from equine spleen (443 kDa), β‐Amylase from sweet potato (200 kDa), bovine serum albumin (BSA, 66 kDa), carbonic anhydrase from bovine erythrocytes (29 kDa), and cytochrome C from horse (12.9 kDa) with respective elution peaks at 11.32, 12.94, 14.84, 17.57, and 18.85 mL. The void volume was determined by dextran blue (2000 kDa) and estimated to be 8.2 mL. The MW estimation of the different MmpL10 variants was done through a calibration curve obtained by plotting the *K*_av_ of each standard protein as a function of the Log_10_ of their MW. The calculation was calculated as follows: *K*_av_ = (*V*_*e*_ − *V*_0_)/(*V*_*t*_ − *V*_0_), with *V*_*e*_ = elution volume of each protein, *V*_0_ = void volume, *V*_*t*_ = 23.56 mL (total CV as given by the manufacturer).

### Negative staining: grid preparation, data collection, and processing

The LMNG condition was chosen as the most suitable for negative staining electron microscopy. Directly after SEC, 3 μL of the top peak fraction of MmpL10‐mEGFP‐FL_Msm_ at a concentration of 0.5 μm was applied on a Formvar/Carbon 200 Mesh (FCF200‐CU‐50) copper grid previously glow discharged for 25 s at 15 mA and 0.39 mBar (PELCO easiGlow; Ted Pella Inc., Redding, CA, USA). After 3 min of incubation at room temperature (RT), the drop was dried with a Whatman paper and stained with 9 μL uranyl acetate 1%. A similar step of drying staining was applied again.

After 1 min of incubation, uranyl acetate was dried, and the grid was left for at least 5 min at RT before storage. Negative staining microscopy was performed using a (JEOL (EUROPE) SAS, Croissy sur Seine, France) 1400 Flash transmission electron microscope at 120 kV equipped with a (GATAN, Pleasanton, CA, USA) One View camera. Sixty‐six micrographs were collected with a pixel size of 2.2 Å, a spherical aberration of 3.4 mm, and a total exposure dose of 40 e^−^·Å^−2^. The 66 micrographs obtained were imported as negative staining data on cryosparc v4.6.2 and estimated with ctffind4 parameters. Sixty‐two micrographs were selected for processing, and 127 230 particles were template‐free picked with a diameter between 80 and 250 Å. Twenty‐two thousand five hundred eighty‐two particles were extracted with a box size of 400^2^ pixels and underwent 2D‐classification. The 10 best classes (12 898 particles) were selected to generate three *ab‐initio* 3D‐reconstructions with a C1 symmetry. Particles used to generate the best volume underwent another round of 2D‐classification, were selected, and were used to generate another *ab‐initio* 3D‐reconstruction (4010 particles, C1 symmetry). The *ab‐initio* 3D‐reconstruction was used in the following homogeneous and nonuniform refinements, resulting in the final EM map. The molecular model generated by alphafold v3 was fit in the EM map using the chimerax software [23].

### Purification of soluble periplasmic extension, CC_Msm_

The plasmid encoding the MmpL10 periplasmic extension pET‐SUMO::CC_Msm_ was transformed into *E. coli* BL21::*pRARE2* strain on an LB plate supplemented with kanamycin (Kan) and chloramphenicol (Cm) antibiotics at 50 and 30 μg·mL^−1^, respectively. A 400 mL LB Kan + Cm preculture was used to start a 12 L LB Kan + Cm culture grown at 37 °C. When OD_600_ reached 0.6–0.8, bacteria were cold‐shocked in an ice bucket for 30 min, and expression of the protein was induced with 1 mm IPTG, and the culture was further grown overnight at 18 °C. Bacteria were harvested, resuspended in lysis buffer, lysed by sonication, and cellular debris was discarded by centrifugation as previously described (cf. MmpL10‐mEGFP‐FL_Msm_ expression and purification). The supernatant was incubated for 1 h at 4 °C under stirring with 6 mL of Nickel‐Nitrilotriacetic acid (Ni‐NTA) Sepharose beads (Cytiva), previously equilibrated with lysis buffer supplemented with 20 mm imidazole. The solution was charged twice into gravity columns and washed once with lysis buffer + 20 mm imidazole, and a second time with 50 mm Tris‐HCl pH 8, 1 M NaCl, 10% glycerol, and 0.5 mm β‐mercaptoethanol. Proteins were eluted with three CV of 50 mm Tris‐HCl pH 8, 200 mm NaCl, 10% glycerol, 0.5 mm β‐mercaptoethanol, and 250 mm imidazole, and dialyzed overnight at 4 °C against 1 L of 50 mm Tris‐HCl pH 8, 200 mm NaCl, 10% glycerol, and 0.5 mm β‐mercaptoethanol. During dialysis, the protein was cleaved by mixing the solution with TEV protease added at a ratio of 1 : 50 w/w. The cleaved protein was charged again on Ni‐NTA beads and collected in the flow‐through. The solution was dialyzed overnight against 1 L of 50 mm Tris‐HCl pH 8, 10% glycerol, and 0.5 mm β‐mercaptoethanol, and charged the day after on a HiTrap Q FF anion exchange chromatography column (Cytiva). Elution was performed with a 20 CV NaCl gradient from 0 to 500 mm with buffers in 50 mm Tris‐HCl pH 8, 10% glycerol, and 0.5 mm β‐mercaptoethanol. The peak fractions were pooled and concentrated in a 10 kDa cutoff Centricon (Sartorius) to 5 mg·mL^−1^ and finally purified by SEC on a Superdex 200 10/300 GL increase column (Cytiva) at a flow rate of 0.35 mL·min^−1^ with 20 mm Tris‐HCl pH 8, 200 mm NaCl, 10 % glycerol, and 1 mm β‐mercaptoethanol.

### Structure predictions

The sequence of all the targeted proteins was submitted to alphafold v2 through the Cosmic2 portal [24] or alphafold server (https://golgi.sandbox.google.com/) running alphafold v3. All the proteins were modeled as monomers and multimers. Structures were visualized and analyzed using the chimerax software.

## Results and Discussion

### Construct design, expression test, and detergent screening

**Fig. 1:**
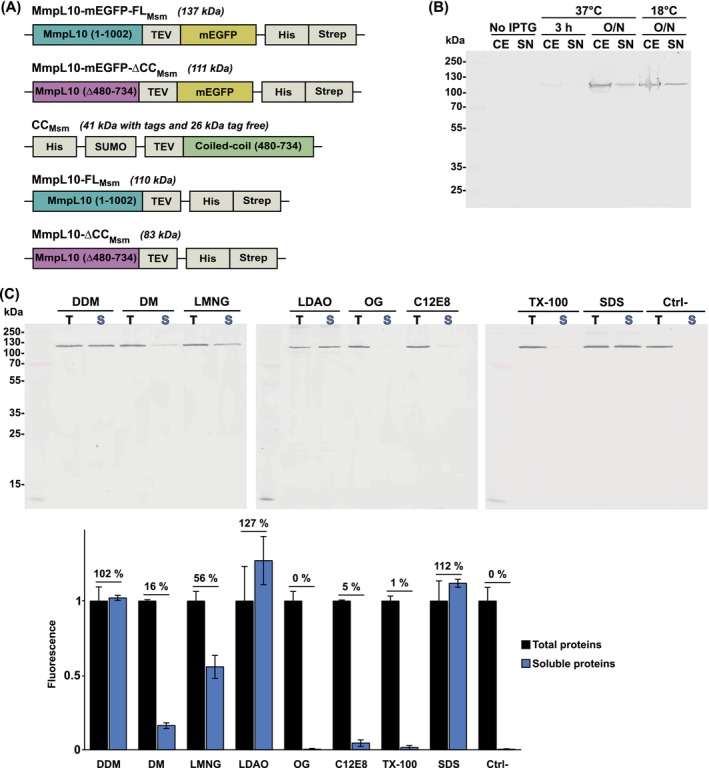
Construct designs, expression, and solubilization optimizations of MmpL10. (A) Schematic of the constructs used to express recombinant MmpL10 from *Msm* (*MSMEG_0410*) and their calculated molecular weights. (B) Expression tests of the MmpL10‐mEGFP‐FL_Msm_ construct (*n* = 1). Comparison of the expression conditions in *E. coli* C43 ∆*acrB*: 3 h at 37 °C, overnight at 37 °C, and overnight at 18 °C. CE: crude extract obtained after lysis, SN: supernatant remaining in the soluble fraction following centrifugation of the lysate. CE and SN were charged on a 10% SDS/PAGE. The bands corresponding to MmpL10 were revealed by in‐gel fluorescence and detected as mentioned below. (C) Screen to identify suitable detergents for solubilization of MmpL10‐mEGFP‐FL_Msm_. The gels are representative of two independent experiments (*n* = 2). Protein was extracted in dodecyl‐β‐d‐maltoside (DDM), *n*‐decyl‐beta‐maltoside (DM), lauryl maltose neopentyl glycol (LMNG), lauryldimethylamine‐N‐oxide (LDAO), OG, C12E8, and TX‐100 detergents for 2 h at 4 °C. SDS was used as a positive control, and a negative control (Ctrl−) without any detergent was also performed in the same conditions. Samples were compared before (T: Total proteins) and after ultracentrifugation (S: Soluble proteins) on a 12% SDS/PAGE. The error bars indicate standard deviation (SD). Fluorescence was visualized with a Li‐Cor scanner using odyssey software and quantified with imagej. For each condition, data were normalized to the amount of fluorescence obtained after extraction, before ultracentrifugation.

To study MmpL10, we employed a heterologous expression strategy in *E*. *coli*. Similar to our previous strategy to express and purify MmpL3 from *Msm* [25], the *MSMEG_0410* gene was synthesized with optimized codons for *E. coli* expression. Following the *mmpL10* coding sequence, we added a sequence coding for a TEV protease cleavage site and a coding sequence for the mEGFP protein (monomeric enhanced green fluorescent protein), a monomeric variant of GFP [26]. Finally, we added nucleotides sequences encoding for a multi‐histidine and a *Strep*‐Tag II tags interspaced by linker regions to enable affinity chromatography purification (Fig. 1A). The mEGFP tag was added to enable fluorescence detection of the protein (Fig. 1A), as we anticipated a low expression yield. The final construct was named MmpL10‐mEGFP‐FL_Msm_.

Next, we screened for optimal protein expression conditions in *E. coli* by assessing expression at different temperatures and/or induction times. As shown in Fig. 1B, similar results were obtained in terms of expression level and solubility after separation of soluble and insoluble fractions by ultracentrifugation. We decided to express the protein at 18 °C for large‐scale expression as the ratio between insoluble and soluble protein was slightly better at this temperature.

Search for an optimal detergent is key to investigating membrane proteins. We therefore performed a detergent screen on *E. coli* membranes to identify the most effective one for MmpL10‐mEGFP‐FL_Msm_ membrane extraction. As shown in Fig. 1C, in‐gel fluorescence quantification attests that out of the eight detergents tested, three (DDM, LDAO, and LMNG) efficiently extracted the protein in a soluble form after ultracentrifugation. DDM and LDAO solubilized all the protein, similar to the SDS positive control, whereas LMNG solubilized approximately half of it.

### MmpL10 forms large oligomers in solution

**Fig. 2:**
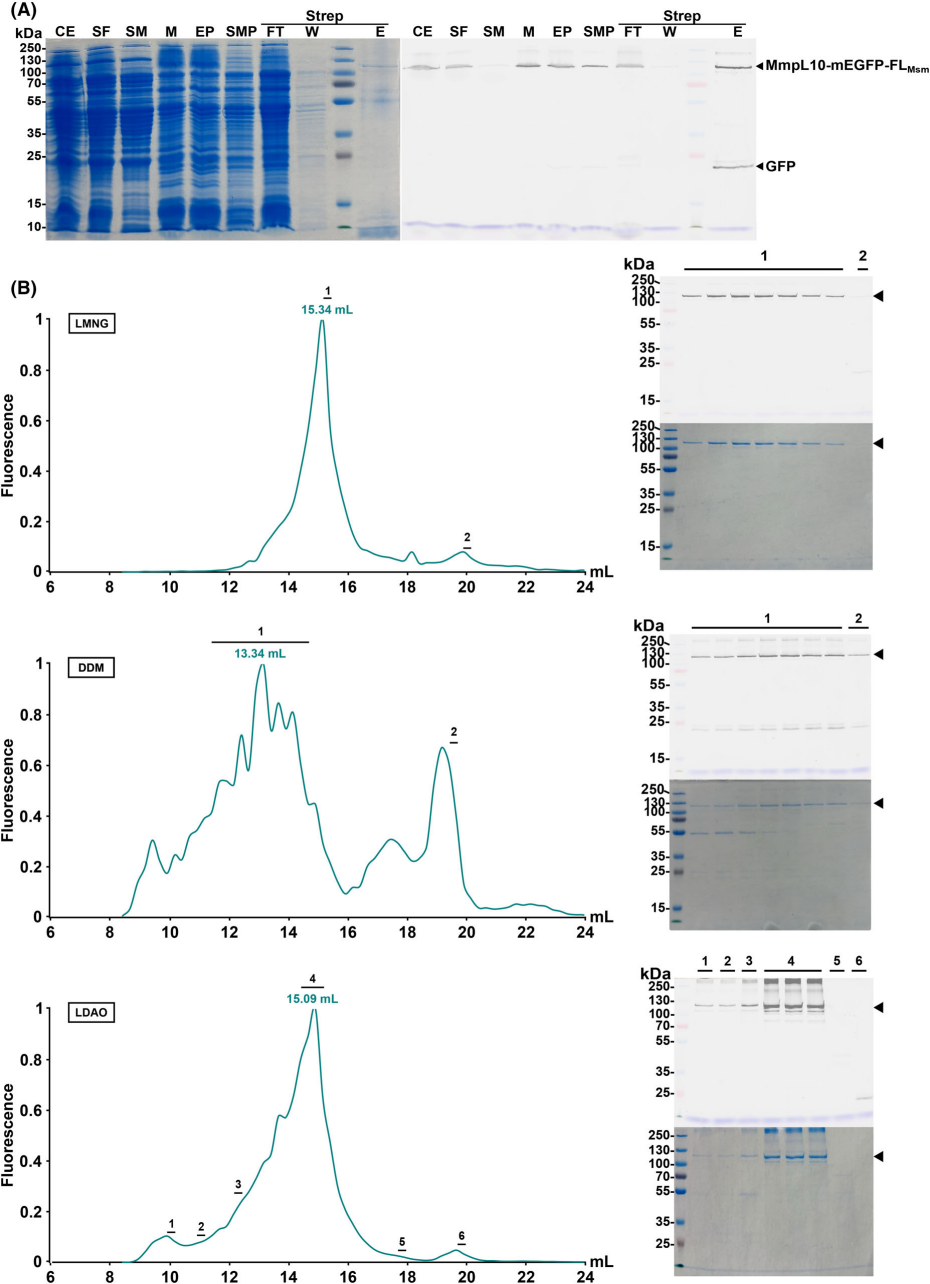
Purification of the MmpL10‐mEGFP‐FL_Msm_ construct. (A) Representative purification out of three independent experiments of the MmpL10‐mEGFP‐FL_Msm_ on affinity chromatography using lauryl maltose neopentyl glycol (LMNG). Proteins were visualized on SDS/PAGE 12%, after Coomassie blue staining (left panel) and using in‐gel fluorescence assessed with a Li‐Cor scanner (right panel). CE: crude extract obtained after lysis, SF: soluble fractions remaining in the supernatant of the centrifuged lysate, SM: soluble material remaining in the supernatant after the first ultracentrifugation, M: membrane pellets resuspended after the first ultracentrifugation, EP: extracted proteins after 2 h stirring with LMNG, SMP: solubilized membrane proteins remaining in the supernatant after the second ultracentrifugation, Strep: *Strep*‐Tactin affinity chromatography (FT: flow‐through, W: wash, and E: elution). (B) Comparison of the size‐exclusion chromatography (SEC) profiles in LMNG (top), dodecyl‐β‐d‐maltoside (DDM) (middle), or lauryldimethylamine‐N‐oxide (LDAO) (bottom) detergents. The chromatograms are representative of 3, 2, and 1 independent experiments, respectively, for LMNG, DDM, and LDAO. Eluted proteins were followed by fluorescence measurement with a Tecan Infinite 200Pro plate reader, and values were normalized to ease the comparison between the different detergents.

To select the most stabilizing MmpL10‐mEGFP‐FL_Msm_ detergent(s), we performed large‐scale expression, followed by purification of MmpL10‐mEGFP‐FL_Msm_ in the presence of DDM, LMNG, or LDAO. After solubilizing the protein, an affinity chromatography step was performed using a *Strep*‐Tactin column, allowing purification to near homogeneity of MmpL10‐mEGFP‐FL_Msm_ with the three detergents (Figs 2A and S1). Since we were unable to remove the tags with the TEV protease, we used the full‐length tagged protein for subsequent characterization. We further purified MmpL10‐mEGFP‐FL_Msm_ by SEC employing a Superpose 6 10/300 GL Increase column. MmpL10‐mEGFP‐FL_Msm_ behaves similarly with the three detergents. It eluted as a protein with a higher MW than expected for a monomeric protein, with elution peaks at 15.34, 13.34, and 15.09 mL in LMNG, DDM, and LDAO, respectively (Fig. 2B). The sharpest and most homogeneous peak was observed with LMNG, and hence, we continued our investigations using this detergent.

### Structural modeling suggests that MmpL10 can form a homotrimer

**Fig. 3:**
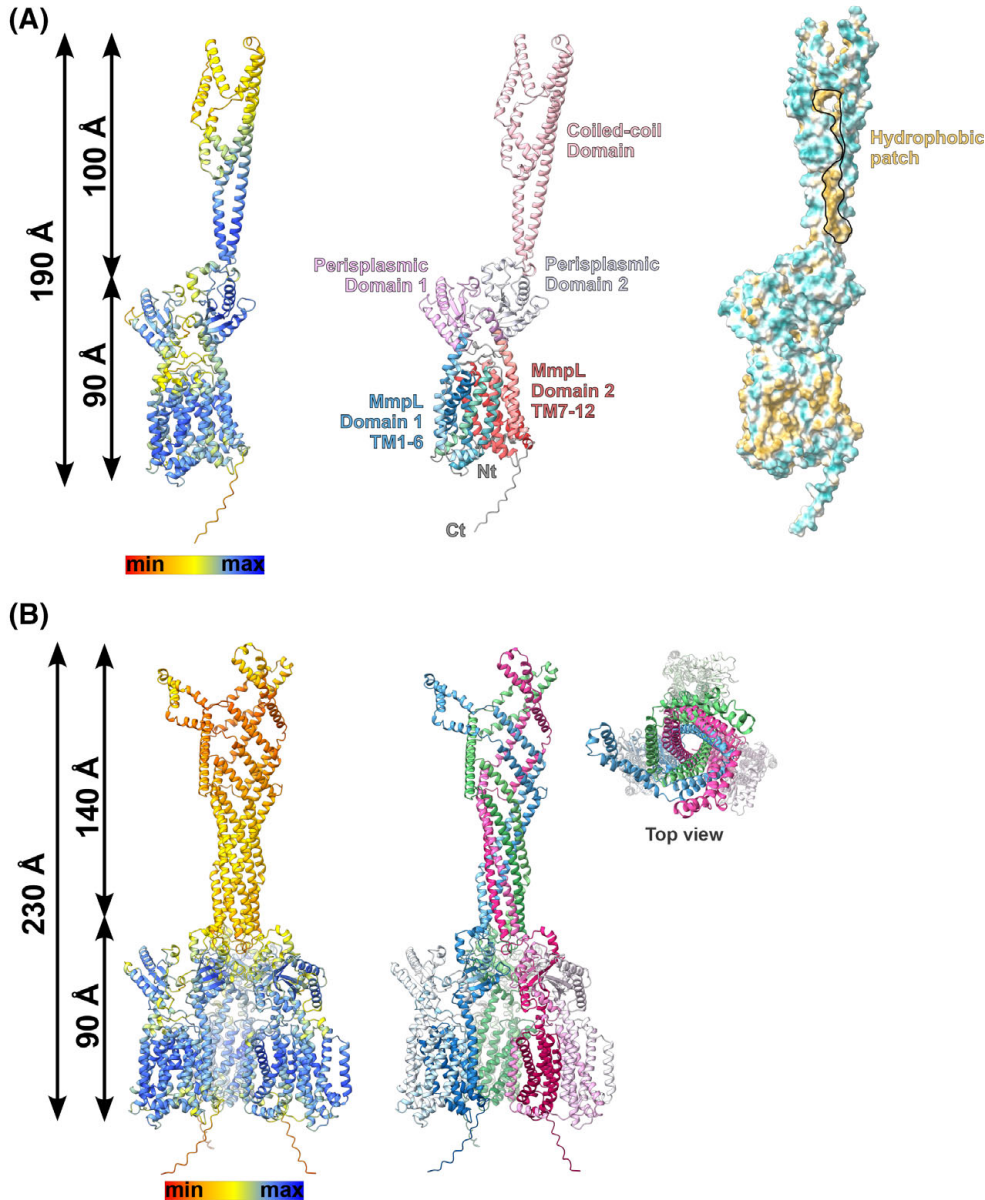
Structures prediction of MmpL10_Msm_. (A) The predicted model of the monomer form of MmpL10_Msm_ is shown as a cartoon (left). The confidence score from alphafold v2 is indicated. The middle panel shows the same model with its domains annotated. The right panel shows the surface representation of MmpL10 where the hydrophilic and neutral amino acids are represented in green and white while the hydrophobic residues are in yellow. (B) Prediction of the MmpL10_Msm_ homotrimer and colored according to the AlphaFold scoring. The three coiled‐coil domains form a tube‐like structure. In the trimer form, the extension is extended by about 40 Å. The right panel displays the same model but colored by chains and a top view of the channel is presented.

As the MmpL oligomeric state is still an open question, we explored the possibility that MmpL10 could form oligomer(s) as suggested by the SEC experiments. *In silico* modeling was used to model MmpL10 as a monomer and trimer. We generated a model of MmpL10_Msm_ in its monomeric state using alphafold version 2 [27]. The structure was predicted with a high confidence score, suggesting an overall shape reminiscent of those of the experimental structures of MmpL3, MmpL5, and MmpL4 (Fig. 3A). The protein is made of two MmpL domains (residues 1 : 345 and 373 : 983), both composed of six transmembrane (TM) helices. A linker region (residues 346–372) interconnects the two transmembrane domains, and the presence of a small unstructured C terminus is predicted. Between the first and second TM helix of each MmpL domain, a periplasmic domain is inserted. While the periplasmic domain (residues 50–173) emerging from the first MmpL domain resembles those described in other MmpL structures, the extension of the second MmpL domain (residues 409–805) is atypical, forming a long stretch that spans residues 480 to 734. This fully helical domain exhibits structural similarities with coiled‐coil domains, which are known to facilitate homo‐ or hetero‐oligomerization, as, for example, observed in TolC [17]. Supporting the notion that this piece partakes in a higher oligomeric assembly, 128 of 255 are hydrophobic residues. Among these, some are involved in the formation of a large hydrophobic patch (Fig. 3A) unlikely to be exposed to a hydrophilic environment, such as the periplasmic space.

Encouraged by these modeling approaches and our SEC data, we next performed the modeling of a homotrimer of MmpL10_Msm_ using alphafold model version 2. The choice of this oligomeric state was driven by the fact that RND family members often organize as homotrimers. Indeed, the alphafold scoring of such a homotrimer was also of high confidence (Fig. 3B). Importantly, the oligomer assembly seems to be mainly driven by the coiled‐coil domain of the three monomers that are intertwined with each other. Few inter‐monomer contacts between other parts of the protein were detected (not shown). Further, the coiled‐coil domain is extended in the trimeric assembly as compared to the monomeric form (140 Å vs. 100 Å, respectively).

### Deletion of the periplasmic extension abolishes MmpL10 oligomerization

**Fig. 4:**
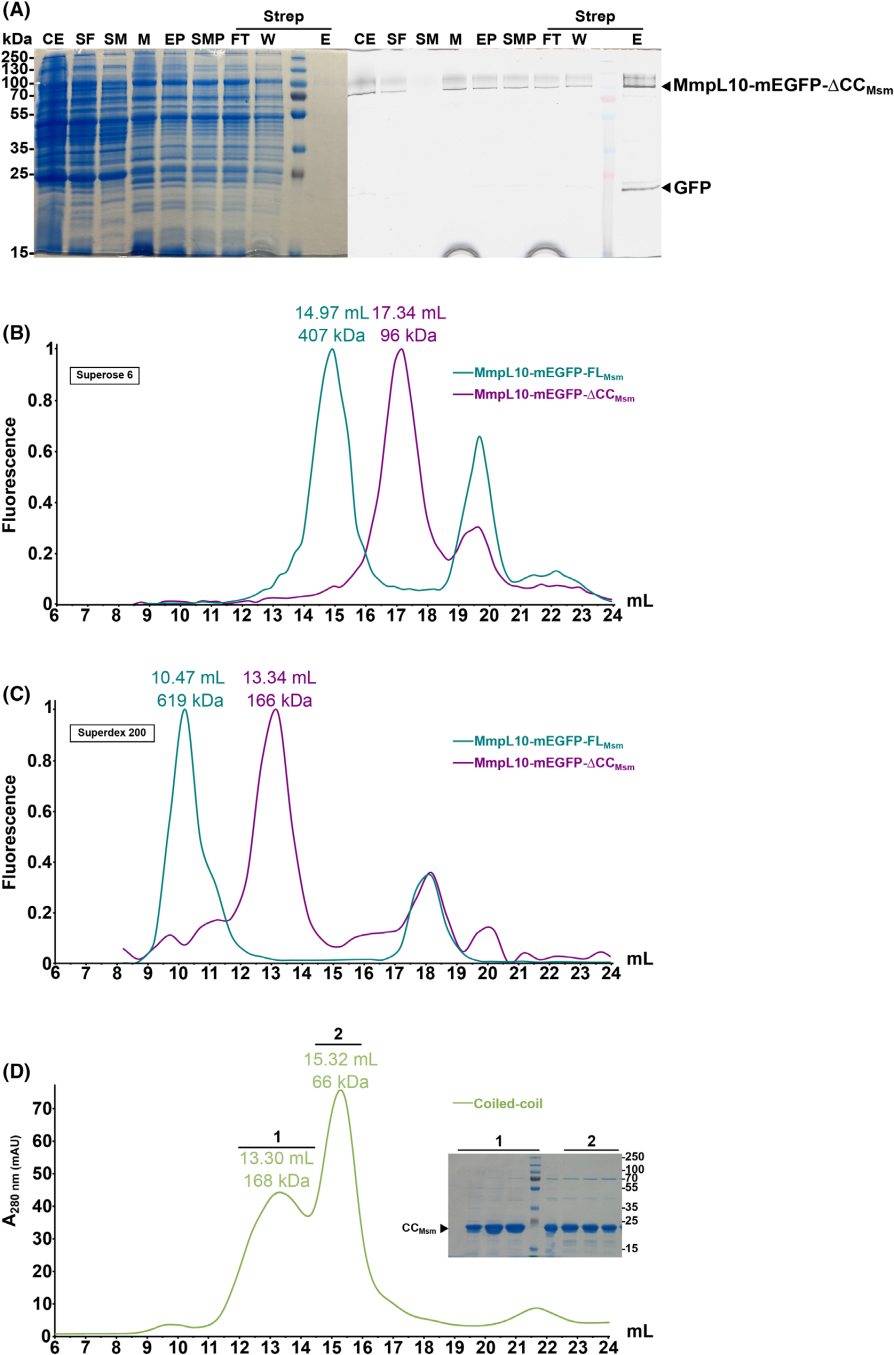
Purification of MmpL10‐mEGFP‐ΔCC_Msm_ and CC_Msm_ variants. (A) Affinity chromatography of the MmpL10‐mEGFP‐ΔCC_Msm_ in lauryl maltose neopentyl glycol (LMNG) (*n* = 1). Proteins were visualized on SDS/PAGE 12%, after Coomassie blue staining (left panel) and in‐gel fluorescence was assessed with a Li‐Cor scanner (right panel). CE: crude extract obtained after lysis, SF: soluble fractions remaining in the supernatant of the centrifuged lysate, SM: soluble material remaining in the supernatant after the first ultracentrifugation, M: membrane pellets resuspended after the first ultracentrifugation, EP: extracted proteins after 2 h stirring with LMNG, SMP: solubilized membrane proteins remaining in the supernatant after the second ultracentrifugation, Strep: *Strep*‐Tactin affinity chromatography (FT: flow‐through, W: wash, and E: elution). (B and C) Comparison of the elution profiles on Superose 6 and Superdex 200 10/300 GL increase columns. The normalized fluorescence of MmpL10‐mEGFP‐FL_Msm_ (cyan) and MmpL10‐mEGFP‐ΔCC_Msm_ (purple) both purified in LMNG, is indicated on the *y*‐axis. The elution volumes of each peak and calculated apparent molecular weights are indicated and were obtained from the calibration curves made using the two columns as described in Materials and methods. Chromatograms in B are representative of three independent experiments for MmpL10‐mEGFP‐FL_Msm_, while gel‐filtration for MmpL10‐mEGFP‐ΔCC_Msm_ was performed once. In C, gel‐filtration was performed once for MmpL10‐mEGFP‐FL_Msm_, and the experiments were repeated three times for MmpL10‐mEGFP‐ΔCC_Msm_. (D) Elution profile of the coiled‐coil domain CC_Msm_ on Superdex 200 10/300 GL increase column. The elution volumes of the peaks and calculated apparent molecular weights are indicated. The chromatogram shown is representative of results obtained from two independent experiments.

To explore whether oligomerization was triggered by the coiled‐coil domain, as suggested by our modeling approach, we generated a truncated version of MmpL10‐mEGFP‐FL_Msm_ named MmpL10‐mEGFP‐∆CC_Msm_ (Fig. 1A), which lacks residues 480–734, corresponding to the predicted coiled‐coil domain. We could express and purify this truncated version (Fig. 4A). The deletion variant MmpL10‐mEGFP‐∆CC_Msm_ eluted significantly later than the wild‐type MmpL10‐mEGFP‐FL_Msm_ on a Superose 6 10/300 GL Increase column with an elution peak at 17.34 mL as compared to that of the wild‐type at 14.97 mL (Fig. 4B).

**Fig. 5:**
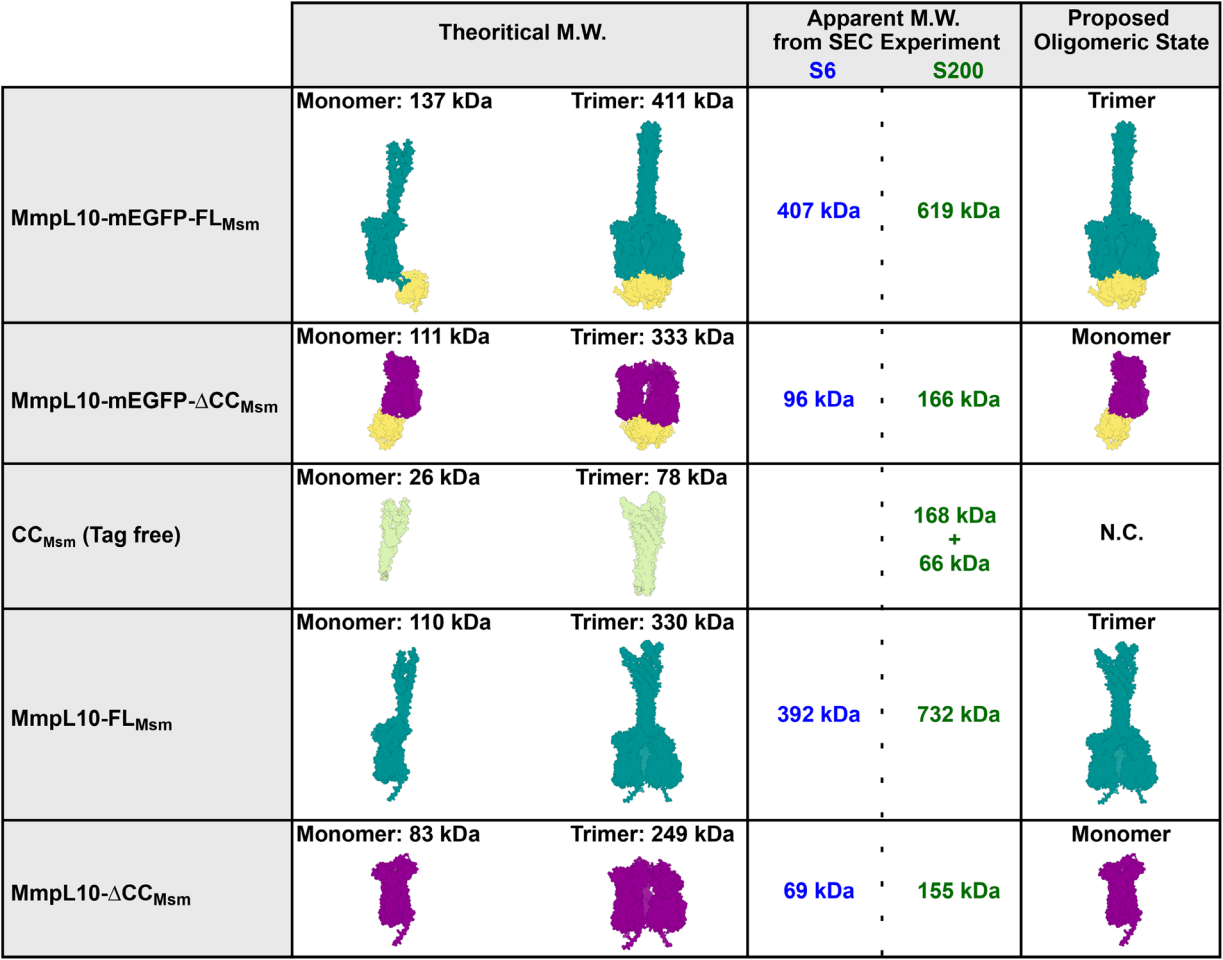
Comparison of the theoretical molecular weights with the apparent molecular weights deduced from size‐exclusion chromatography of the MmpL10 constructs. The figure summarizes all the results of this study in terms of molecular weight and oligomeric state estimation. All the models presented are labeled similarly as in Fig. 1: MmpL10 full‐length in cyan, MmpL10 without coiled‐coil domain in magenta, coiled‐coil domain alone in light green, and mEGFP in yellow. All the molecular weights indicated are either theoretical or calculated from the protein sequence of the constructs only, and calculated from the calibration curves of the selected SEC column. S6 and S200 indicate Superose 6 and Superdex 200 SEC columns. N.C. stands for nonconclusive as we could not assign the oligomeric state of the coiled‐coil domain. The schematic representations of the structures were generated with chimerax.

Based on a column calibration curve, we estimated the apparent MW of MmpL10‐mEGFP‐FL_Msm_ to be about 407 kDa, while its theoretical mass is 137 kDa (Fig. 5). In contrast, the apparent mass of the truncated form was estimated to be about 96 kDa as compared to its theoretical MW of 111 kDa. These data strongly suggest that the wild‐type form is a trimer as its theoretical MW is 411 kDa, while the mutant is monomeric (Fig. 5). We further confirmed this oligomeric state difference by loading the samples on another SEC column, the Superdex 200 10/300 GL column (Fig. 4C) that gives a more precise mass estimation for proteins of mass near 100 kDa. The elution shift between the two protein forms was confirmed as the mutant version was eluted at 13.34 mL (apparent MW 166 kDa), which was about 3 mL after the wild‐type (apparent MW 619 kDa) (Figs 4C and 5).

It is worth mentioning that we did not consider the presence of the LMNG belt in our theoretical calculation of the MWs of the different proteins. The MWs presented in Fig. 5 were calculated based solely on the protein sequence. Furthermore, similarly, we did not apply any correction to account for the presence of a detergent crown when reporting the apparent MWs in solution, as deduced from the SEC experiments. This could explain why, for instance, the experimentally determined apparent MW of 166 kDa for MmpL10‐mEGFP‐∆CC_Msm_ is larger than the theoretical mass of 111 kDa. The 55 kDa difference is thus likely due to the presence of an LMNG detergent belt and also because SEC does not allow determining absolute MW but only apparent MW.

Finally, analysis of the structure predictions identified a few inter‐monomer contacts between the transmembrane domains of the three protomers of the trimer model (not shown), suggesting that the trimer formation is mainly mediated by the coiled‐coil domain. To corroborate this hypothesis, we assessed whether this domain could oligomerize on its own. To this end, the coiled‐coil domain (residues 480–734) was expressed fused to a His and a SUMO tags (Fig. 1A), purified (Fig. S2), and loaded on a Superdex 200 Increase 10/300 GL column (Fig. 4D). The final tagged free version was named CC_Msm_ (Figs 1A and 5). CC_Msm_ was eluted as two different peaks at 13.3 and 15.3 mL on SEC corresponding to apparent MW of 168 and 66 kDa, respectively, and as compared to a calculated mass of 26 kDa. The estimation of the mass by SEC could not be determined precisely as the overall shape of the domain is not expected to be globular; however, its elution as two distinct and well‐separated peaks is consistent with the formation of oligomers (Fig. 5).

**Fig. 6:**
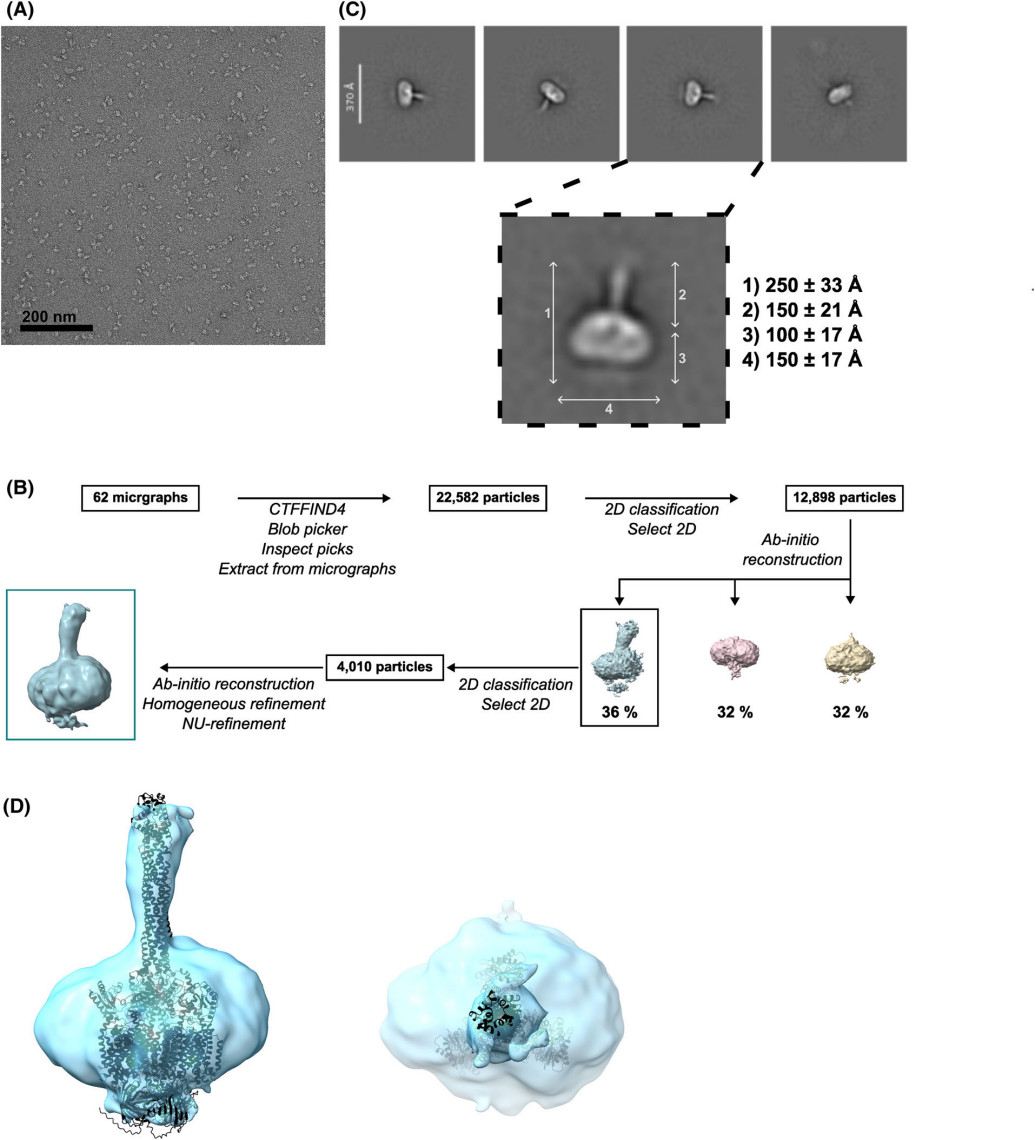
Investigation of MmpL10 by negative staining electron microscopy. (A) One representative micrograph of the 62 acquired of purified protein in lauryl maltose neopentyl glycol (LMNG) and at 0.5 μm. Two datasets from two different protein preparations were collected with similar results. The scale bar represents 200 nm. (B) Overview of the data processing steps on cryosparc. NU indicates nonuniformed. (C) 2D‐classification averages of the final map and mean dimensions of the measured particles. Fifty particles (10 particles from five different micrographs) were measured; the standard deviation is indicated. The scale bar indicates 370 Å. (D) alphafold v3 model of MmpL10‐mEGFP‐FL_Msm_ fitted into the obtained EM map and shown as side and top views.

Importantly, the MmpL10‐mEGFP‐FL_Msm_ construct possesses a large tag in its C terminus, which may contribute to or disturb oligomerization. To rule out this possibility, we made two new constructs expressing the full‐size MmpL10 (MmpL10‐FL_Msm_) or its coiled‐coil depleted form (MmpL10‐∆CC_Msm_), fused to His and *Strep*‐Tag II (FL) tags in their C terminus (Fig. 1A). Using the same purification protocol as the mEGFP forms, we confirmed that MmpL10‐FL_Msm_ can form oligomers that are coiled‐coil dependent with elution volumes of 15.02 mL (392 kDa) and 10.09 mL (732 kDa) on Superose 6 and Superdex 200 10/300 GL Increase columns, while the truncated form MmpL10‐∆CC_Msm_ eluted at 17.90 mL (69 kDa) on the Superose 6 column and 13.45 mL (155 kDa) on the Superdex 200 column (Figs 5 and S3). It is important to note that when comparing the elution peaks of MmpL10‐mEGFP‐FL_Msm_ versus the one of MmpL10‐FL_Msm_, we expected a delay in elution between the two forms. However, despite the fact that mEGFP adds about 80 kDa to the trimer construct, both MmpL10‐mEGFP‐FL_Msm_ and MmpL10‐FL_Msm_ eluted similarly from the Superose 6 column (14.97 mL vs. 15.02 mL). A plausible explanation based on model predictions is that the mEGFP is very close to the MmpL10 part and does not seem to trigger a significant extension of the MmpL10 structure (Figs 5 and 6). Thus, the overall shape of the mEGFP construct is likely not much different from the untagged version. This could explain why both MmpL10 versions behave similarly in solution during SEC, which separates proteins according to their overall shape.

Collectively, our biochemical data and modeling approaches strongly suggest that MmpL10_Msm_ can form large oligomers compatible with a trimer and that this oligomerization is triggered by the coiled‐coil domain.

### Negative stained electron microscopy supports the homotrimer hypothesis

To support these findings, we performed negative stain electron microscopy on MmpL10‐mEGFP‐FL_Msm_ purified in LMNG. Particles were sufficiently homogeneous for further analysis as shown on a representative micrograph (Fig. 6A). A large number of particles displayed an ‘Erlenmeyer’‐like shape (Fig. 6A–C), with mean dimensions similar to those displayed by our alphafold generated structure: 250 Å for the longest distance, a width of 100 Å, compatible with MmpL10 in a detergent micelle, and a length for the extruding tube of about 150 Å (Fig. 6C). Importantly, we also observed similar particles with protein purified in DDM instead of LMNG, indicating that the shape was not inherent to the selected detergent (not shown).

We attempted to reconstruct a three‐dimensional volume of MmpL10‐mEGFP‐FL_Msm_ and succeeded in obtaining a low‐resolution EM map (Fig. 6B), in which we could fit the trimeric alphafold v3 model of MmpL10‐mEGFP‐FL_Msm_ (Fig. 6D). Thus, the EM map supported the presence of a MmpL10 trimer embedded in a detergent belt, with a somewhat bent tube‐like extension formed by three coiled‐coil domains.

### The coiled‐coil domain is widely distributed in mycobacterial MmpLs and may contribute to ligand export

The data presented above highlight the role of the coiled‐coil domain in MmpL10 oligomerization. To explore whether this domain is present in other MmpLs and may contribute to their oligomerization, we modeled all MmpL proteins from *Msm* as monomers and trimers. The presence of this domain was detected in all MmpLs except in MmpL3 and MmpL11, and could play a crucial role in the oligomerization of all of these transporters (Fig. S4). Moreover, we observed that this domain is present in MmpL proteins from most mycobacteria and particularly the well‐studied ones, such as *Mtb* or *Mab* (not shown). It has been reported that deletion of the coiled‐coil domain in MmpL10 from *Mab* abrogates TPP precursor transport [20], while its removal in MmpL4 and MmpL5 abolished mycobactin and bedaquiline efflux [16]. These studies, in conjunction with ours, suggest that, like other RND proteins, such as the tripartite AcrAB‐TolC complex, most MmpLs need to oligomerize for their transport/efflux function [17].

**Fig. 7:**
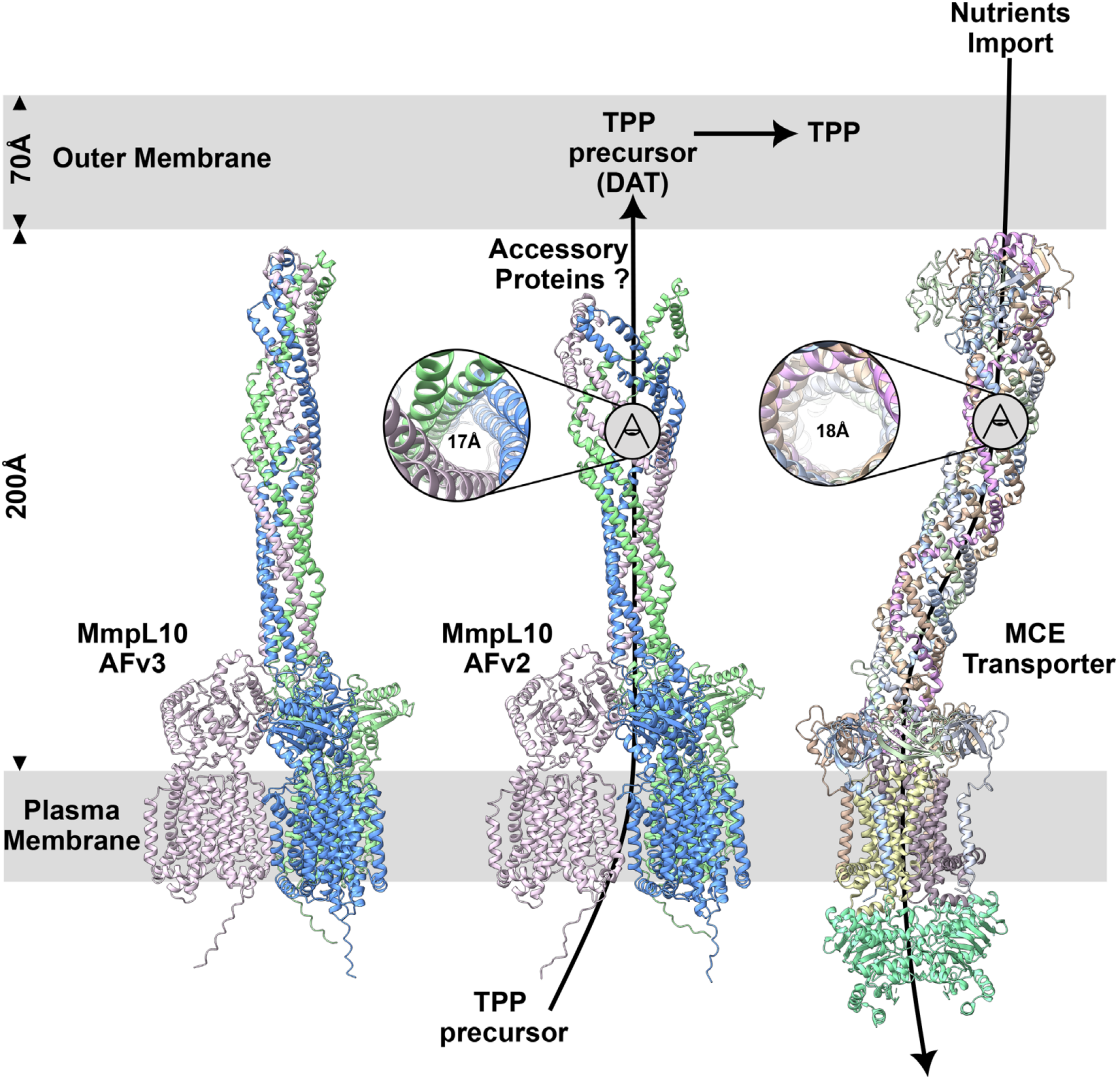
Comparison of the MmpL10 trimer and the MCE lipid transporter from mycobacteria. The mammalian cell entry (MCE) transporter structure corresponds to PDB accession 8fef. Both MmpL10 models obtained either with alphafold v2 (AFv2) or v3 (AFv3) are displayed and colored by chains. The distance of the different mycobacterial membrane compartments is indicated. Top views in both channel sections are indicated next to each structure as well as the approximate size of the tube.

The tube‐like structure of the MmpL10 periplasmic extension also brings to mind the recently determined structure of the MCE lipid transporter [28]. This ABC transporter, which imports nutrients through the thick mycobacterial envelope, is elongated by a ring‐like structure formed by several Mce proteins that stack together to form a needle‐like channel structure. Interestingly, the authors reported densities in the EM map that are compatible with the existence of large lipids, such as mycolic acids in the channel. Although our structural data are preliminary, they support that a long channel‐like structure could be formed by the coiled‐coil domain in MmpL10 and potentially in other MmpL proteins. The central and hydrophobic cavity of the tunnel is as large as the one formed by Mce proteins. Considering that the length of this tube‐like structure may extend from the inner membrane and potentially reach (monomeric model) or even span (trimeric model) the outer membrane, and given its diameter close to the one seen in the MCE lipid transporter, we hypothesize that it could play a key role in the transport of the MmpL ligands to the mycomembrane (Fig. 7). A high‐resolution structure of a MmpL trimer with bound ligands would be extremely useful to validate this model.

## Author contributions

JC and MB contributed to the conceptualization, methodology, investigation, EM data analysis, structure modeling, and original draft writing. JF contributed to the investigation. JC, CC, KB, PG, and MB contributed to the data analysis. AA and JLKH assisted with EM data acquisition. PG, CC, and MB contributed to the funding acquisition and project administration. All the authors were involved in reviewing and editing the manuscript.

## Conflict of interest

The authors declare no conflict of interest.

## Peer review

The peer review history for this article is available at https://www.webofscience.com/api/gateway/wos/peer‐review/10.1002/1873‐3468.70085.

## Supporting information


**Fig. S1.** Purification of MmpL10‐mEGFP‐FL_Msm_ in DDM and LDAO detergents.
**Fig. S2**. Purification of CC_Msm_.
**Fig. S3**. Purification of MmpL10‐FL_Msm_ and MmpL10‐∆CC_Msm_ in LMNG.
**Fig. S4**. Structure predictions of all MmpL proteins from *Mycobacterium smegmatis* mc^2^‐155.

## Data Availability

The data that support the findings of this study are available from the corresponding author mickael.blaise@umontpellier.fr upon reasonable request.

## References

[1] Viljoen A , Dubois V , Girard‐Misguich F , Blaise M , Herrmann J‐L and Kremer L (2017) The diverse family of MmpL transporters in mycobacteria: from regulation to antimicrobial developments. Mol Microbiol 104, 889–904. doi: 10.1111/mmi.13675 28340510

[2] Domenech P , Reed MB and Barry CE (2005) Contribution of the *Mycobacterium tuberculosis* MmpL protein family to virulence and drug resistance. Infect Immun 73, 3492–3501. doi: 10.1128/IAI.73.6.3492-3501.2005 15908378 PMC1111821

[3] Zhang B , Li J , Yang X , Wu L , Zhang J , Yang Y , Zhao Y , Zhang L , Yang X , Yang X *et al*. (2019) Crystal structures of membrane transporter MmpL3, an anti‐TB drug target. Cell 176, 636–648.e13. doi: 10.1016/j.cell.2019.01.003 30682372

[4] Su C‐C , Klenotic PA , Bolla JR , Purdy GE , Robinson CV and Yu EW (2019) MmpL3 is a lipid transporter that binds trehalose monomycolate and phosphatidylethanolamine. Proc Natl Acad Sci USA 116, 11241–11246. doi: 10.1073/pnas.1901346116 31113875 PMC6561238

[5] Xu Z , Meshcheryakov VA , Poce G and Chng S‐S (2017) MmpL3 is the flippase for mycolic acids in mycobacteria. Proc Natl Acad Sci USA 114, 7993–7998. doi: 10.1073/pnas.1700062114 28698380 PMC5544280

[6] Stevens CM , Babii SO , Pandya AN , Li W , Li Y , Mehla J , Scott R , Hegde P , Prathipati PK , Acharya A *et al*. (2022) Proton transfer activity of the reconstituted *Mycobacterium tuberculosis* MmpL3 is modulated by substrate mimics and inhibitors. Proc Natl Acad Sci USA 119, e2113963119. doi: 10.1073/pnas.2113963119 35858440 PMC9335285

[7] Babii S , Li W , Yang L , Grzegorzewicz AE , Jackson M , Gumbart JC and Zgurskaya HI (2024) Allosteric coupling of substrate binding and proton translocation in MmpL3 transporter from *Mycobacterium tuberculosis* . MBio 15, e02183‐24. doi: 10.1128/mbio.02183-24 39212407 PMC11481577

[8] Su C‐C , Klenotic PA , Cui M , Lyu M , Morgan CE and Yu EW (2021) Structures of the mycobacterial membrane protein MmpL3 reveal its mechanism of lipid transport. PLoS Biol 19, e3001370. doi: 10.1371/journal.pbio.3001370 34383749 PMC8384468

[9] Adams O , Deme JC , Parker JL , CRyPTIC Consortium , Fowler PW , Lea SM *et al*. (2021) Cryo‐EM structure and resistance landscape of *M. tuberculosis* MmpL3: an emergent therapeutic target. Struct Lond Engl 1993 29, 1182–1191. doi: 10.1016/j.str.2021.06.013 PMC8752444 34242558

[10] Couston J , Guo Z , Wang K , Gourdon P and Blaise M (2023) Cryo‐EM structure of the trehalose monomycolate transporter, MmpL3, reconstituted into peptidiscs. Curr Res Struct Biol 6, 100109. doi: 10.1016/j.crstbi.2023.100109 38034087 PMC10682824

[11] Maharjan R , Zhang Z , Klenotic PA , Gregor WD , Tringides ML , Cui M , Purdy GE and Yu EW (2024) Structures of the mycobacterial MmpL4 and MmpL5 transporters provide insights into their role in siderophore export and iron acquisition. PLoS Biol 22, e3002874. doi: 10.1371/journal.pbio.3002874 39423221 PMC11524445

[12] Maharjan R , Zhang Z , Klenotic PA , Gregor WD , Purdy GE and Yu EW (2024) Cryo‐EM structure of the *Mycobacterium smegmatis* MmpL5–AcpM complex. MBio 15, e03035‐24. doi: 10.1128/mbio.03035-24 39480109 PMC11633376

[13] Cioccolo S , Barritt JD , Pollock N , Hall Z , Babuta J , Sridhar P , Just A , Morgner N , Dafforn T , Gould I *et al*. (2024) The mycobacterium lipid transporter MmpL3 is dimeric in detergent solution, SMALPs and reconstituted nanodiscs. RSC Chem Biol 5, 901–913. doi: 10.1039/d4cb00110a 39211474 PMC11352979

[14] Belardinelli JM , Yazidi A , Yang L , Fabre L , Li W , Jacques B , Angala , Rouiller I , Zgurskaya HI , Sygusch J *et al*. (2016) Structure‐function profile of MmpL3, the essential mycolic acid transporter from *Mycobacterium tuberculosis* . ACS Infect Dis 2, 702–713. doi: 10.1021/acsinfecdis.6b00095 27737557 PMC5117480

[15] Yamamoto K , Nakata N , Mukai T , Kawagishi I and Ato M (2021) Coexpression of MmpS5 and MmpL5 contributes to both efflux transporter MmpL5 Trimerization and drug resistance in *Mycobacterium tuberculosis* . mSphere 6, e00518‐20. doi: 10.1128/mSphere.00518-20 33408221 PMC7845600

[16] Earp JC , Garaeva AA , Meikle V , Niederweis M and Seeger MA (2025) Structural basis of siderophore export and drug efflux by *Mycobacterium tuberculosis* . Nat Commun 16, 1934. doi: 10.1038/s41467-025-56888-6 39994240 PMC11850643

[17] Alav I , Kobylka J , Kuth MS , Pos KM , Picard M , Blair JMA and Bavro VN (2021) Structure, assembly, and function of tripartite efflux and type 1 secretion Systems in gram‐negative bacteria. Chem Rev 121, 5479–5596. doi: 10.1021/acs.chemrev.1c00055 33909410 PMC8277102

[18] Wetzel KS , Illouz M , Abad L , Aull HG , Russell DA , Garlena RA , Cristinziano M , Malmsheimer S , Chalut C , Hatfull GF *et al*. (2023) Therapeutically useful mycobacteriophages BPs and muddy require trehalose polyphleates. Nat Microbiol 8, 1717–1731. doi: 10.1038/s41564-023-01451-6 37644325 PMC10465359

[19] Gorzynski M , De Ville K , Week T , Jaramillo T and Danelishvili L (2023) Understanding the phage‐host interaction mechanism toward improving the efficacy of current antibiotics in *Mycobacterium abscessus* . Biomedicine 11, 1379. doi: 10.3390/biomedicines11051379 PMC10216323 37239050

[20] Malmsheimer S , Daher W , Tasrini Y , Hamela C , Aguilera‐Correa JJ , Chalut C , Hatfull GF and Kremer L (2024) Trehalose polyphleates participate in *Mycobacterium abscessus* fitness and pathogenesis. MBio 15, e0297024. doi: 10.1128/mbio.02970-24 39475242 PMC11633156

[21] Burbaud S , Laval F , Lemassu A , Daffé M , Guilhot C and Chalut C (2016) Trehalose polyphleates are produced by a glycolipid biosynthetic pathway conserved across phylogenetically distant mycobacteria. Cell Chem Biol 23, 278–289. doi: 10.1016/j.chembiol.2015.11.013 27028886

[22] Thouvenel L , Prevot G , Chiaradia L , Parra J , Mouton‐Barbosa E , Locard‐Paulet M , Marcoux J , Tropis M , Burlet‐Schiltz O , Daffé M *et al*. (2020) The final assembly of trehalose polyphleates takes place within the outer layer of the mycobacterial cell envelope. J Biol Chem 295, 11184–11194. doi: 10.1074/jbc.RA120.013299 32554804 PMC7415978

[23] Meng EC , Goddard TD , Pettersen EF , Couch GS , Pearson ZJ , Morris JH and Ferrin TE (2023) UCSF chimerax: tools for structure building and analysis. Protein Sci 32, e4792. doi: 10.1002/pro.4792 37774136 PMC10588335

[24] Cianfrocco MA , Wong‐Barnum M , Youn C , Wagner R and Leschziner A (2017) COSMIC2: a science gateway for Cryo‐electron microscopy structure determination. Pract Exp Adv Res Comput 1–5. doi: 10.1145/3093338.3093390

[25] Ung KL , Alsarraf H , Kremer L and Blaise M (2022) Corrigendum to “MmpL3, the trehalose monomycolate transporter, is stable in solution in several detergents and can be reconstituted into peptidiscs” [Protein Expr Purif 191 (March 2022), 106014]. Protein Expr Purif 195–196, 106088. doi: 10.1016/j.pep.2022.106088 35365368

[26] Zacharias DA , Violin JD , Newton AC and Tsien RY (2002) Partitioning of lipid‐modified monomeric GFPs into membrane microdomains of live cells. Science 296, 913–916. doi: 10.1126/science.1068539 11988576

[27] Jumper J , Evans R , Pritzel A , Green T , Figurnov M , Ronneberger O , Tunyasuvunakool K , Bates R , Žídek A , Potapenko A *et al*. (2021) Highly accurate protein structure prediction with alphafold . Nature 596, 583–589. doi: 10.1038/s41586-021-03819-2 34265844 PMC8371605

[28] Chen J , Fruhauf A , Fan C , Ponce J , Ueberheide B , Bhabha G and Ekiert DC (2023) Structure of an endogenous mycobacterial MCE lipid transporter. Nature 620, 445–452. doi: 10.1038/s41586-023-06366-0 37495693 PMC12817965

