# Supplementary material for: A coiled‐coil domain triggers oligomerization of MmpL10, the mycobacterial transporter of trehalose polyphleate precursor
Source: FEBS Lett. 2025 Jun 4;599(12):1682–97. doi: 10.1002/1873-3468.70085 (PMC12183618; doi:10.1002/1873-3468.70085)
Supplement: Supplementary file 1 — Fig. S1. Purification of MmpL10‐mEGFP‐FLMsm in DDM and LDAO detergents. Fig. S2. Purification of CCMsm. Fig. S3. Purification of MmpL10‐FLMsm and MmpL10‐∆CCMsm in LMNG. Fig. S4. Structure predictions of all MmpL proteins from Mycobacterium smegmatis mc2‐155. [file FEB2-599-1682-s001.pdf]

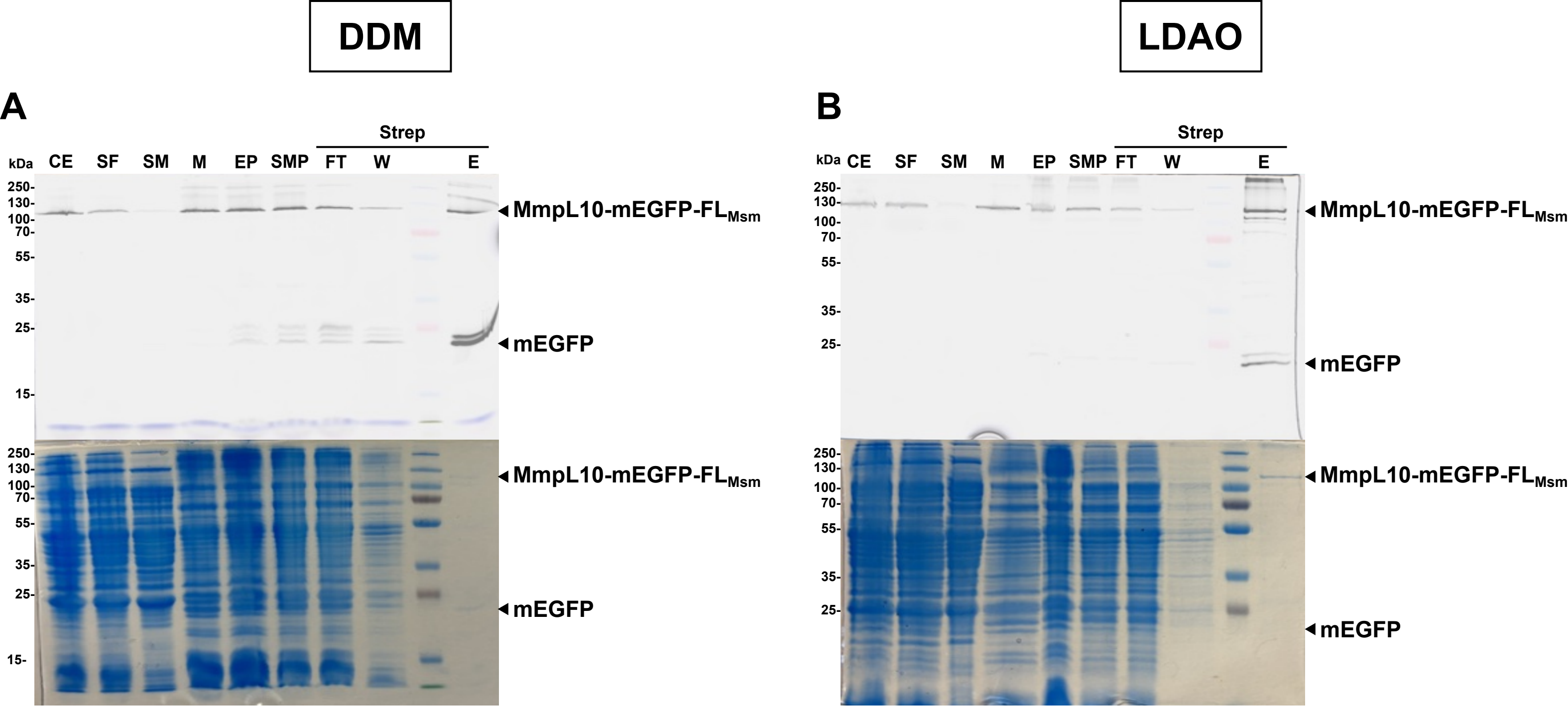

Supplementary Figure 1.

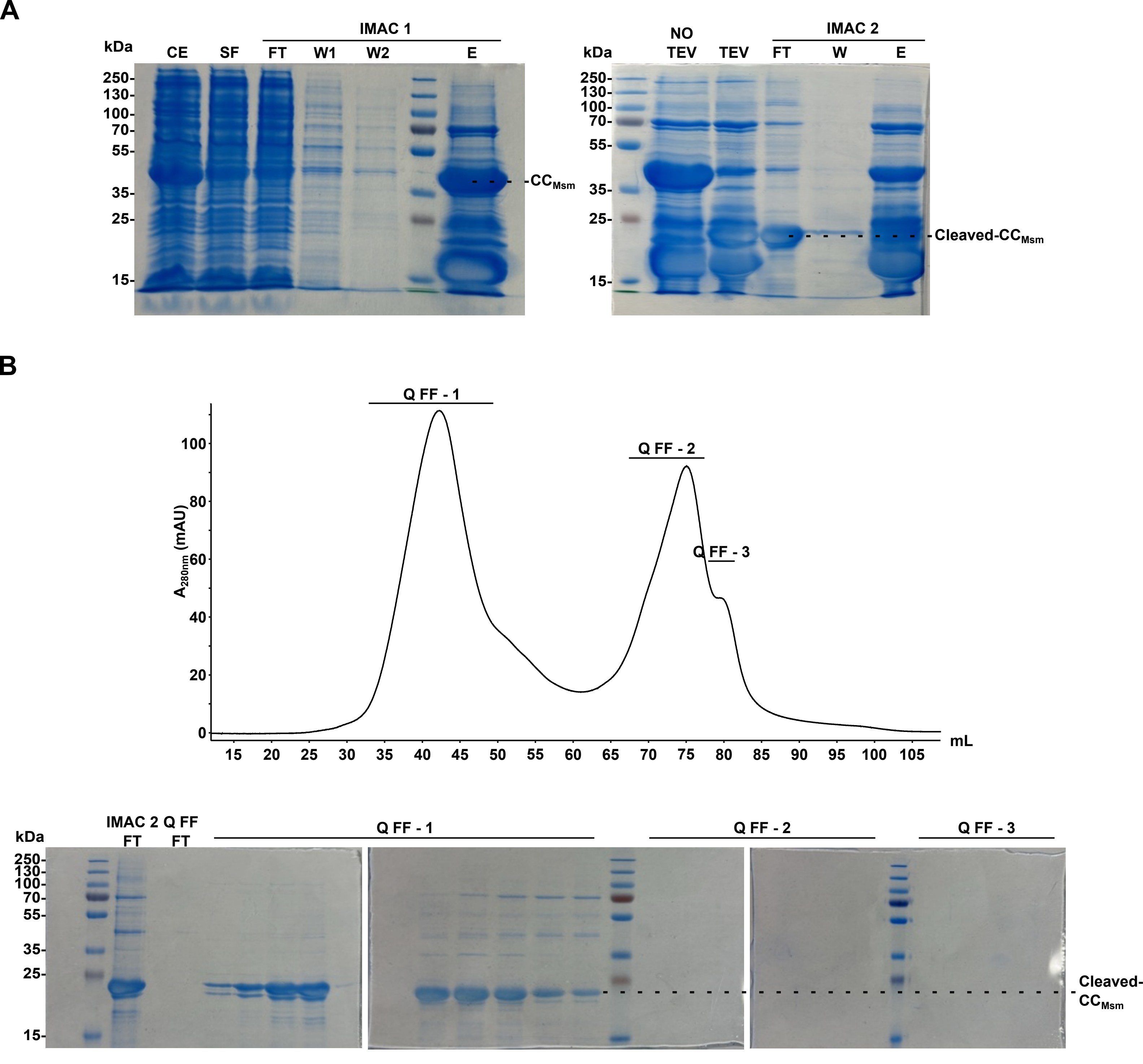

Supplementary Figure 2.

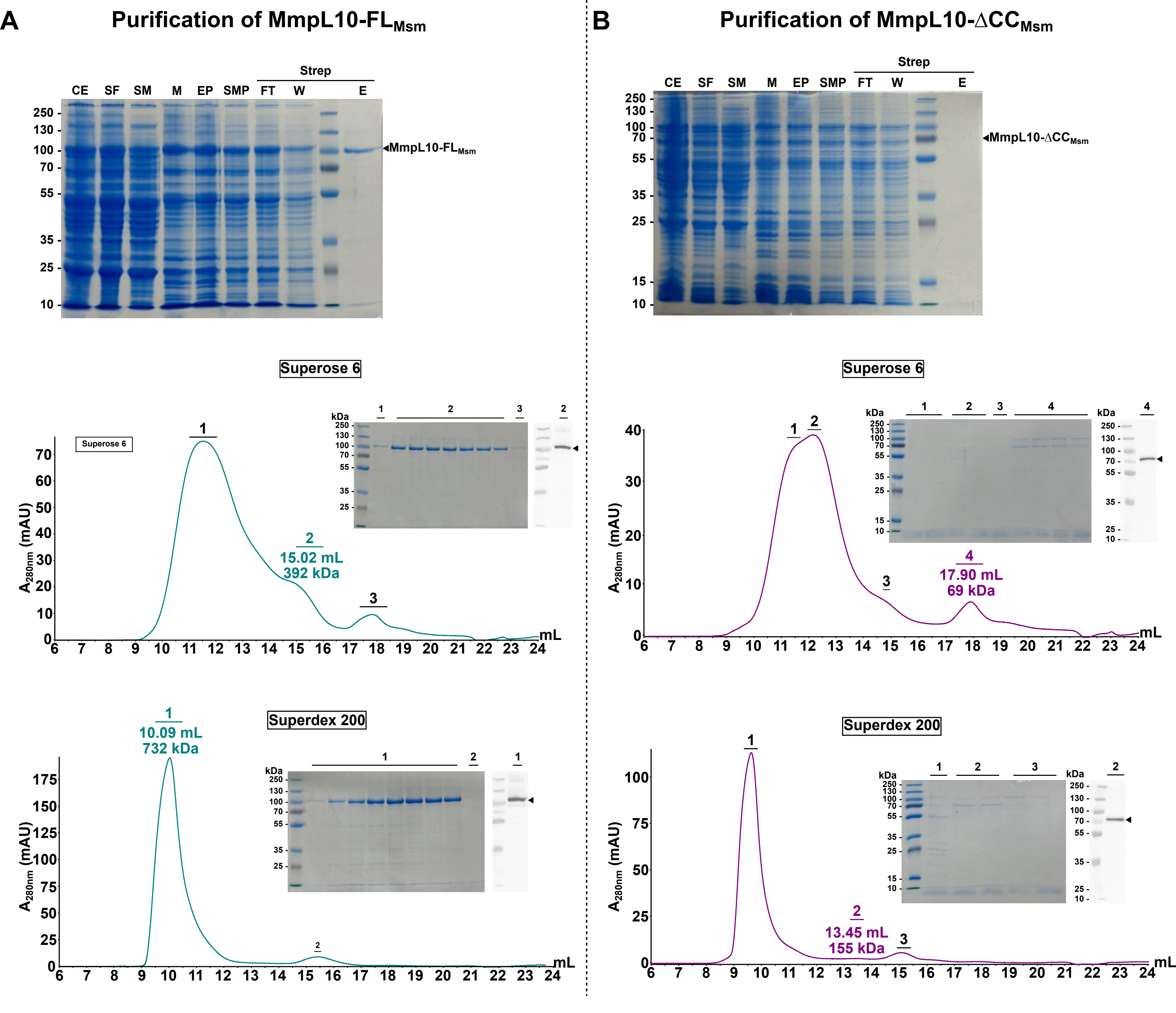

Supplementary Figure 3.

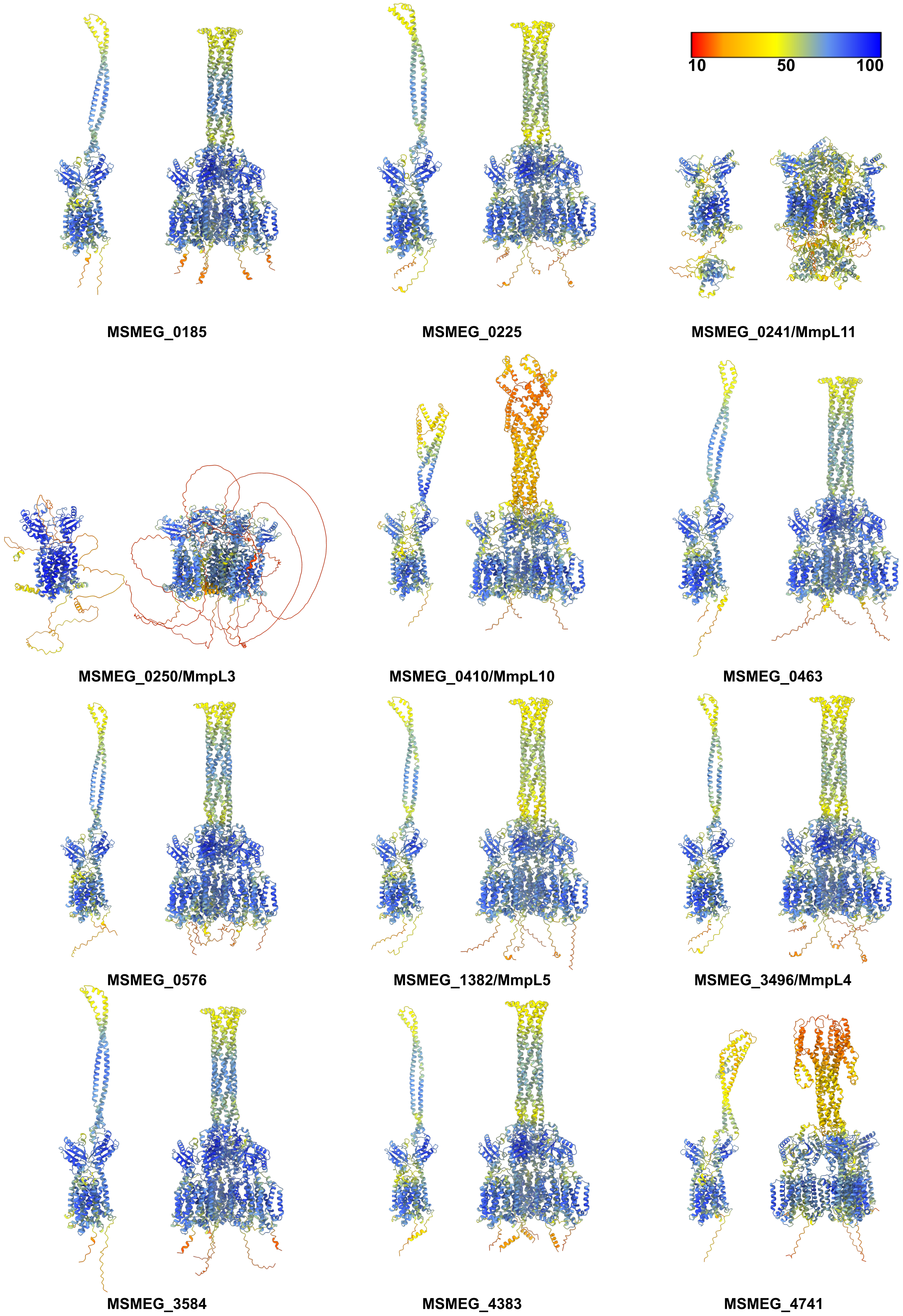

Supplementary Figure 4.
